# Angiogenic Potential of Endothelial Cells in Response to the Stiffness and Anisotropy of Right Ventricle Mimetic Scaffolds

**DOI:** 10.3390/bioengineering13080849

**Published:** 2026-07-23

**Authors:** Yuecheng Wang, Michael Nguyen-Truong, Raghavan Chinnadurai, Peiman Hematti, William R. Wagner, Zhijie Wang

**Affiliations:** 1 Department of Biomedical and Chemical Engineering, Colorado State University, Fort Collins, CO 80523, USA; 2 Carle Illinois College of Medicine, University of Illinois, Urbana, IL 61801, USA; 3 Department of Biomedical Sciences, Mercer University School of Medicine, Savannah, GA 31324, USA; 4 Division of Hematology/Oncology, Medical College of Wisconsin, Milwaukee, WI 53226, USA; 5 Department of Bioengineering, University of Pittsburgh, Pittsburgh, PA 15219, USA; 6 Department of Mechanical Engineering, San Diego State University, San Diego, CA 92182, USA

**Keywords:** mechanobiology, endothelial cell, matrix elasticity, cardiac angiogenesis, right heart failure

## Abstract

Right ventricular (RV) failure secondary to pulmonary hypertension manifests in significant biomechanical alterations of myocardial tissue, including capillary rarefaction and enhanced stiffness and anisotropy. However, how the changes in mechanical cues affect the angiogenic potential of endothelial cells (ECs)—and thereby impact the RV failure progression—remains unclear. The aim of this study is to investigate the effects of RV-relevant substrate stiffness and anisotropy on different EC types using polyurethane urea scaffolds engineered to mimic RV tissues. We find that substrate anisotropy increased EC number but reduced metabolic activity in both cell types. In contrast, the two cell types exhibit divergent responses in their angiogenic potential and angiogenic protein secretome. For HUVECs, neovessel formation is suppressed by substrate stiffening and anisotropy, whereas for HCMECs, it is increased by stiffening and suppressed by anisotropy. Our results highlight the mechanobiological regulation of ECs in a tissue- and cell-type-dependent manner, which is critical for new biomaterial or in vitro model development for cardiac diseases.

## 1. Introduction

Right ventricular (RV) failure is a major cause of mortality in patients with pulmonary hypertension and left heart failure with preserved ejection fraction [1]. The diseased RV remodeling is involved with fibrosis and capillary rarefaction, with the latter compromising nutrient and blood supply to the organ and impairing cardiac muscle cells [2,3,4,5,6]. RV capillary density has been shown to decrease in prior preclinical studies of RV failure [6,7,8,9], and it persists throughout the development stages from early adaptation to late maladaptation [10]. To restore RV function, a potential therapeutic strategy is to promote angiogenesis in the myocardium and revive the dysfunctional cardiomyocytes [11,12,13].

To repopulate the endothelial cells (ECs), it is important to take the pathological mechanical conditions of the myocardium into consideration. The RV myocardium exhibits nonlinear, anisotropic hyperelastic or viscoelastic mechanical behavior due to the gradual recruitment of collagen fibers from early to late diastole [14,15,16,17]. With chronic pressure overload, the RV becomes stiffer and more anisotropic [14,15,17,18,19,20]. The alterations in the mechanical environment of the RV can potentially have a profound influence on the restoration of the endothelial cell (EC) population, including the proliferation and growth that are key to angiogenic outcomes.

Prior studies on 2D or 3D substrates have highlighted that ECs are sensitive to substrate stiffness. When cultured on hard polyacrylamide-based substrates (~55 kPa), ECs get elongated and the cell spreading rate increases compared to those cultured on soft substrates (~180 Pa) [21]. Early studies of EC vascularization on collagen gels revealed that increased matrix rigidity leads to larger lumens, and the response is related to RhoA/ROCK-mediated traction forces [22,23]. But the gel stiffness was not quantified, and thus its physiological relevance is unclear. Conversely, studies using hydrogels of varying stiffness from 100 Pa to a few kPa show a general trend of enhanced vascularization in softer matrices (reviewed by Barrasa-Ramos et al. [24]). While angiogenesis is a process that is involved in various physiological and pathological conditions (from development to tumor growth), the roles of matrix stiffness in endothelial behavior may vary in different scenarios (with matrix elasticity ranging from single digits of Pa to hundreds of kPa). For a relatively young area of research like cardiac angiogenesis, the influence of cardiac mechanical properties on EC angiogenic potential has been rarely studied.

In addition to stiffness, substrate anisotropy may influence EC behaviors via various mechanisms. In most studies, the investigation of substrate anisotropy is performed through the adjustment of fiber alignment (random or aligned) in the substrate; thus, this physical cue is sometimes categorized as the topography architecture of the substrate. Generally, fiber alignment induces higher levels of cellular and cytoskeletal alignment and elongation compared to random fibers, as well as increased adhesion and migration (reviewed by Dessalles et al. [25]). In other cases, the ‘anisotropy’ is related to biaxial mechanical stretch/strain experienced by the cells. For instance, cell alignment is found to be stronger under higher anisotropic/biaxial strains [26]. The substrate anisotropy is particularly relevant in a failing RV as the chronic remodeling accentuates fiber alignment and the mechanical anisotropy of the tissue [14,15,17,18,19,20]. Our group has previously fabricated RV-biomimetic scaffold groups that recapitulate the stiffness and anisotropy of native healthy and diseased RVs, and a synergistic effect of stiffness and anisotropy on mesenchymal stromal cells’ pro-angiogenic potential has been reported [27]. However, how EC behavior is affected by RV-like stiffness and anisotropy is unknown.

Lastly, previous work has shown that ECs from distinct origins or anatomical locations respond differently to mechanical signals [24,25,28]. While human umbilical cord endothelial cells (HUVECs) have been extensively studied for their cellular functions and angiogenic capabilities, they do not reside in cardiac tissues. In contrast, human cardiac microvascular endothelial cells (HCMECs) are more relevant to cardiac angiogenesis but have not been well investigated. In addition, it remains unknown whether macrovascular and cardiac microvascular ECs respond differently to substrate mechanical properties.

To address the knowledge gaps mentioned above, this study aims to investigate the angiogenic potential of ECs in response to RV-like substrate stiffness and anisotropy. We hypothesize that substrate stiffening and the presence of anisotropy exert opposing and divergent effects on EC-mediated angiogenesis. We adopted previously developed electrospun polyurethane urea (PEUU) scaffolds with de-coupled stiffness and anisotropy, mimicking the mechanical properties of healthy and failing RV tissue [27]. HUVECs and HCMECs were cultured on these scaffolds and then assessed for cell number, metabolic activity, and angiogenic potential. We found that substrate stiffness and anisotropy differentially regulated EC number, metabolic activity, and angiogenic potential, and their effects were cell-type dependent. These findings are important for understanding EC mechanobiology in the failing RV and developing more effective in vitro models or biomaterials to investigate disease mechanisms and restore cardiac function.

## 2. Methods

### 2.1. Scaffold Preparation, Cell Seeding and Culture

**Figure 1:**
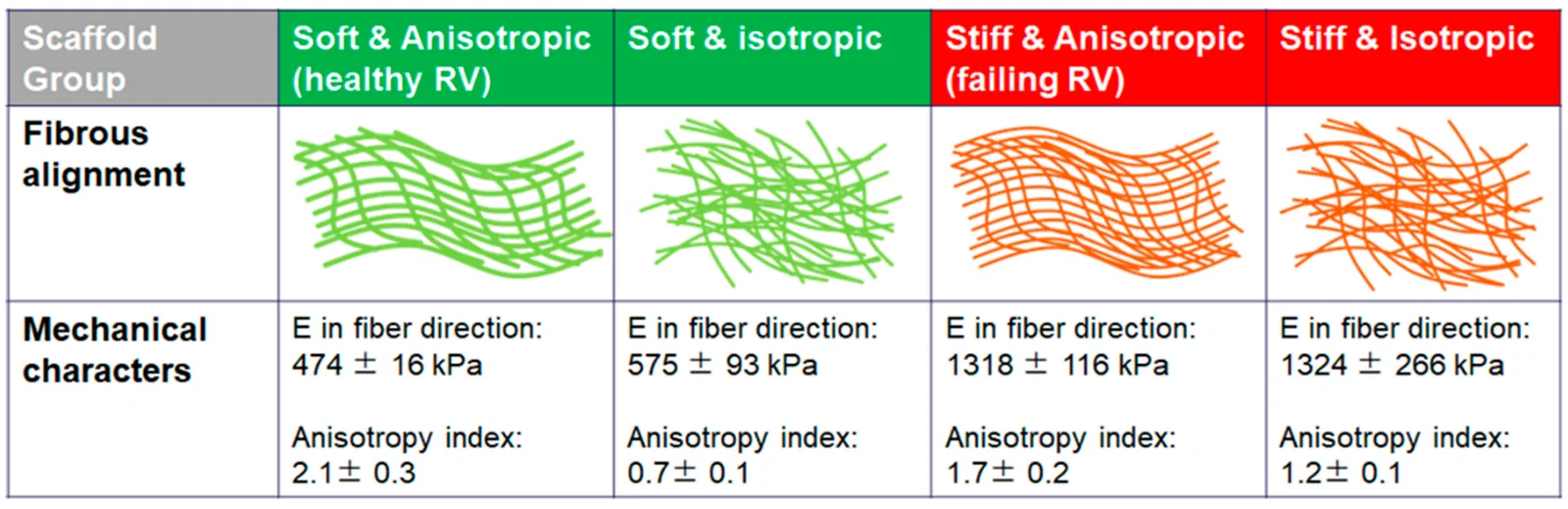
Summary of the PEUU scaffolds used in the study. The elasticity (E: elastic modulus) and anisotropy index (the ratio of E in fiber and cross-fiber directions) were measured by biaxial tensile mechanical tests previously [27].

We have previously developed a tissue-engineered platform to fabricate PEUUs scaffolds with varying stiffness and degrees of anisotropy to mimic the anisotropic elastic properties of normal (soft) and failing (stiff) RVs. Moreover, the scaffolds were fabricated with isotropic and anisotropic patterns by using randomly aligned nanofibers and aligned nanofibers, respectively, to decouple the effect of elasticity and anisotropy [27]. Thus, the four groups of scaffolds used in this study (Figure 1) include: the soft and anisotropic scaffold that represents healthy RV tissue; the stiff and anisotropic scaffold that simulates failing RV tissue; and two additional scaffold groups with identical stiffness but being isotropic. Specifically, the elastic modulus (E) of the healthy RV was approximately 500 kPa, and thus the two soft PEUU scaffold groups had the E of 474 ± 16 kPa and 575 ± 93 kPa, respectively. The elastic modulus (E) of diseased (failing) RV tissue was above 1 MPa, and thus the two stiff PEUU scaffold groups had the E of 1318 ± 116 kPa and 1324 ± 266 kPa, respectively. The degree of anisotropy was chosen in a binary pattern—RV tissue-like anisotropy or isotropy, and thus the anisotropic groups exhibited an anisotropy index of ~2 (2.1 ± 0.2 and 1.7 ± 0.2) whereas the isotropic groups exhibited an anisotropy index of ~1 (i.e., similar elasticity in fiber and cross-fiber directions).

Scaffolds measuring 1 × 1 cm^2^ were attached to the base of 24-well plates by silicone adhesive. The scaffolds were then sterilized by immersion in 70% ethanol for 15 min, followed by three washes with 1× phosphate-buffered saline (PBS) to completely remove residual ethanol. Subsequently, the scaffolds were soaked in 1× PBS for 24 h prior to cell seeding. Next, human umbilical vein endothelial cells (HUVECs, C-12200, PromoCell) and human cardiac microvascular endothelial cells isolated from heart ventricles from a single donor (HCMECs, C-12285, PromoCell, Heidelberg, Germany), at passage 2 to 5, were seeded and cultured in endothelial cell growth medium (CCM029, R&D Systems, Minneapolis, MN, USA) supplemented with 10% (*v*/*v*) fetal bovine serum (FBS) and 1% (*v*/*v*) penicillin-streptomycin. For each scaffold type or control (tissue-culture plate), 2 wells were used to collect results from cell or condition-medium measurements. A total of four independent experiments were performed in this study. Cells were seeded into each well at a density of approximately 5 × 10^4^ cells/cm^2^. The cultures were incubated at 37 °C in a humidified atmosphere with 5% CO_2_ for 72 h. This duration was chosen based on prior papers on similar studies of cell mechanobiology, including ECs and mesenchymal stromal cells [27,29,30].

### 2.2. Metabolic Activity Assay

After 72 h of cell culture, the plates were carefully removed from the incubator for metabolic activity measurements. The standard protocol provided by the kit (DAL1025, Invitrogen, Carlsbad, CA, USA) was adapted. Each well was supplemented with 200 μL of AlamarBlue Cell Viability Reagent. The plates were immediately covered with aluminum foil to protect the reagent from light exposure and returned to the incubator. The incubation was continued under the same conditions (37 °C, 5% CO_2_) for an additional 8 h. Following this incubation period, the conditioned medium from each well, along with protocol-related controls (blank and fully reduced groups), was collected and measured using a plate reader at wavelengths of 570 nm and 600 nm. The metabolic activity of the cells was then calculated using the formula below. Next, the calculated percentage of Alamar Blue reduction was further normalized by the cell number in each sample, yielding a dimensionless value representing metabolic activity per cell (arbitrary units, A.U.) (Equation (1)).

(1) $$Cell\;Metabolic\;Activity\;(\%)=\frac{\left({570\mathrm{nm}}_{experimental}-{600\mathrm{nm}}_{experimental}\right)}{\left({570\mathrm{nm}}_{control}-{600\mathrm{nm}}_{control}\right)}\times 100$$

### 2.3. Cell Number Measurement

Using the same batch of wells, we performed cell counts to quantify EC number. The conditioned medium (CM) and non-adherent (floating) cells were carefully aspirated from the wells, leaving only the scaffolds with adherent cells in the wells. Next, the plate was inverted so that the bottom side with attached cells was imaged by the microscope objective. Brightfield microscope images were captured at 40× magnification, and the total number of cells in the field of view was counted by an observer blinded to the conditions. During the measurement, the scaffolds were kept moist by spraying CM to maintain cell viability.

### 2.4. Tubulogenesis Assay

In a separate set of experiments, a tubulogenesis assay was performed to assess ECs’ angiogenic potential regulated by various scaffolds’ mechanical properties. Briefly, 32 μL of Geltrex (Gibco Geltrex, Reduced Growth Factor Basement Membrane Matrix, Catalog # A1413301; Thermo Fisher Scientific, Waltham, MA, USA) was added into each well in a 96-well plate, followed by solidification for 30 min in an incubator. Approximately 3 × 10^4^ HUVECs or HCMECs were then seeded into each well and incubated with 75 μL of CM collected from the cells previously cultured on various scaffold groups. After 12 h, brightfield images of neovessel formation were captured using an AmScope microscope (AmScope, Irvine, CA, USA) at 40× magnification. Quantification indices such as total tube length, total branching length, number of nodes, and number of branches were obtained using the Angiogenesis Analyzer plugin in ImageJv 1.54 (NIH, Bethesda, MD, USA) as described previously [27].

### 2.5. Endothelial Secretome Profiling Multiplex Array

Pooled conditioned media of three independent experiments were stored at −80 °C. Upon thawing, supernatants were centrifuged at 2000 rpm for 10 min to remove debris. The CM were subsequently analyzed by magnetic bead-based multiplex LuminexTM assays for 12-plex endothelial angiogenic and injury markers which included: E-selectin, P-selectin, vascular endothelial growth factor (VEGF)-A, VEGF-D, platelet endothelial cell adhesion molecule (PECAM)-1, angiopoietin-1, Syndecan-1, VEGF-R2, VEGF-R3, matrix metalloproteinase (MMP)-1, Thrombomodulin (TM), VLA-4 (a receptor for vascular cell adhesion molecule-1). The assay was performed according to the manufacturer’s instructions (ProcartaPlex™ Human Endothelial Injury Marker Panel EPX120-15849-901; Thermo Fisher Scientific, Waltham, MA) using Luminex xMAPTM (multi-analyte profiling) technology. Results were shown as femtograms per milliliter (fg/mL) and then normalized by the total cell numbers.

### 2.6. Statistical Analysis

Data were presented as Mean ± SD and analyzed using GraphPad Prismv10.3.0. A one-way ANOVA with Tukey’s post hoc multiple comparison test was performed for comparisons among the four groups for each cell type, with the significance level set at 0.05.

## 3. Results

### 3.1. Substrate Anisotropy Increases Cell Number in Both Cell Types

**Figure 2:**
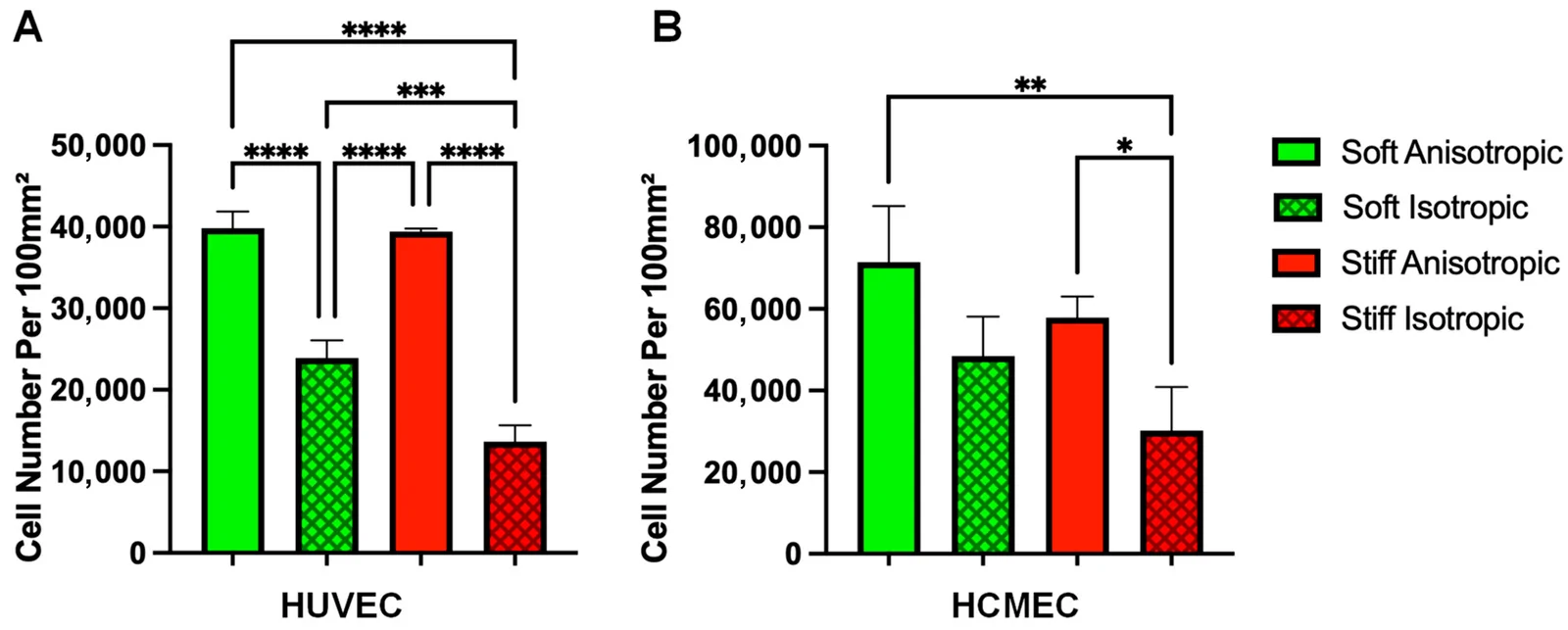
Substrate anisotropy increases cell number as indicated by cell counts of HUVECs (**A**) and HCMECs (**B**) after 72 h of culture on the scaffold groups. * *p* < 0.05, ** *p* < 0.01, *** *p* < 0.005, **** *p* < 0.001.

First, we examined cell number after 72 h of culture on the scaffold groups. As shown in Figure 2, in the scaffolds with similar stiffness (color), the anisotropic substrate (solid bar) led to significantly higher cell numbers than the isotropic substrate (shaded bar), indicating that substrate anisotropy increases cell number. This behavior was observed in both cell types. Furthermore, we found that substrate stiffness does not have a pronounced effect on cell number in either cell type. The only exception is the HUVECs in the isotropic scaffolds, where a reduction in cell number was observed in the stiff group compared to the soft group. Thus, our results indicate that anisotropy plays a more critical and positive role in increasing endothelial cell number than substrate stiffness. Overall, HCMECs had higher cell numbers than HUVECs in each individual group (*p* < 0.05).

### 3.2. Substrate Anisotropy Reduces Cell Metabolic Activity for Both Cell Types

**Figure 3:**
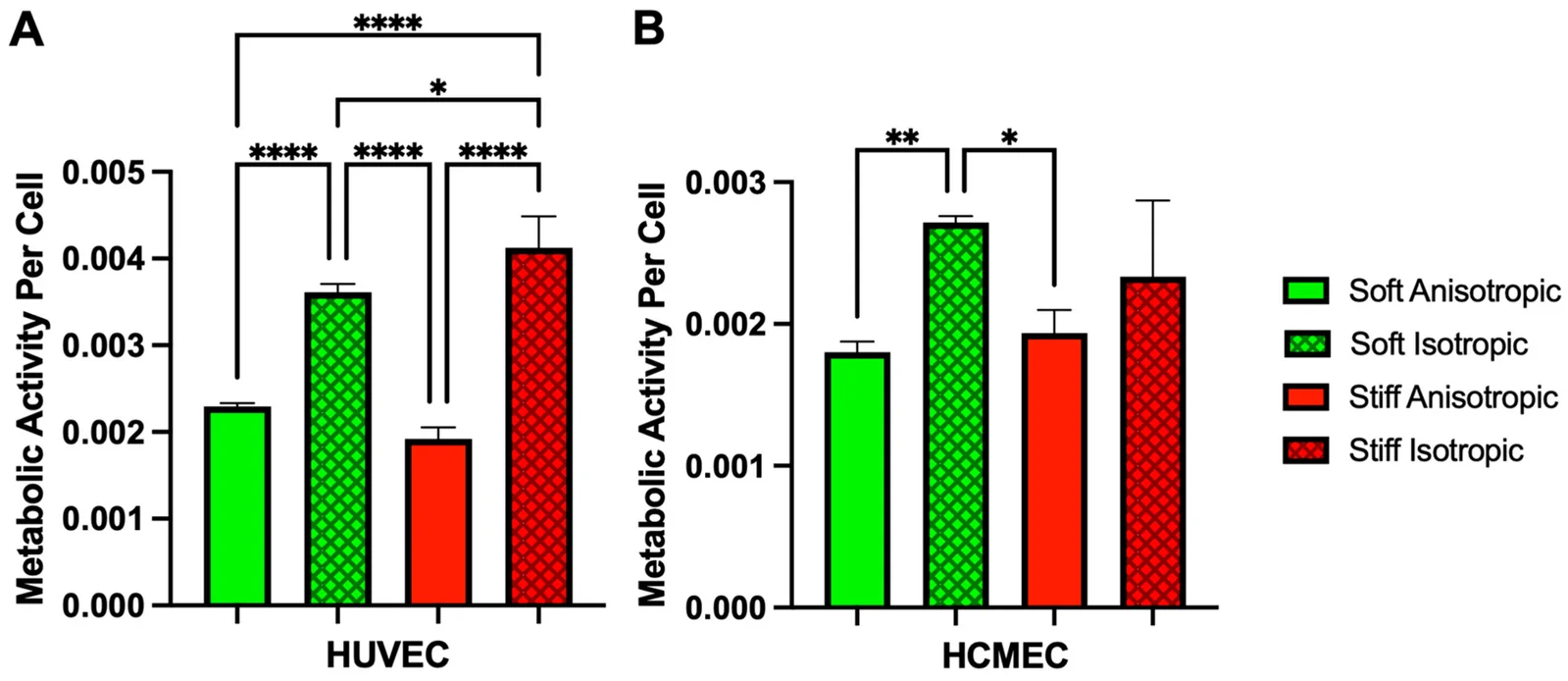
Substrate anisotropy reduces the metabolic activity of HUVECs (**A**) and HCMECs (**B**) after 72 h of culture on the scaffold groups. * *p* < 0.05, ** *p* < 0.01, **** *p* < 0.001.

Next, we measured the metabolic activity of the cells to elucidate the effects of mechanical properties on EC behavior. As we found different cell numbers in various scaffold groups (Figure 2), we present the following results per cell measurement to reflect the individual cell’s behavior regulated by the mechanical environment. As shown in Figure 3, in the scaffold groups with similar stiffness (color), the anisotropic scaffold (solid bar) led to reduced metabolic activity compared to the isotropic scaffold (shaded bar). This behavior was observed for both cell types. This indicates that substrate anisotropy lowers EC metabolic activity under both stiff and soft conditions, and in both cell types. In contrast, we did not observe a significant effect of substrate stiffness on the cell metabolic activity, except that in HUVEC, a stiff and isotropic scaffold resulted in higher metabolic activity than a soft and isotropic scaffold. Thus, our results indicate that anisotropy plays a more critical and negative role in suppressing endothelial cell metabolic activity than substrate stiffness.

### 3.3. Neovessel Formation Is Inhibited by Substrate Stiffening and Anisotropy in HUVEC

**Figure 4:**
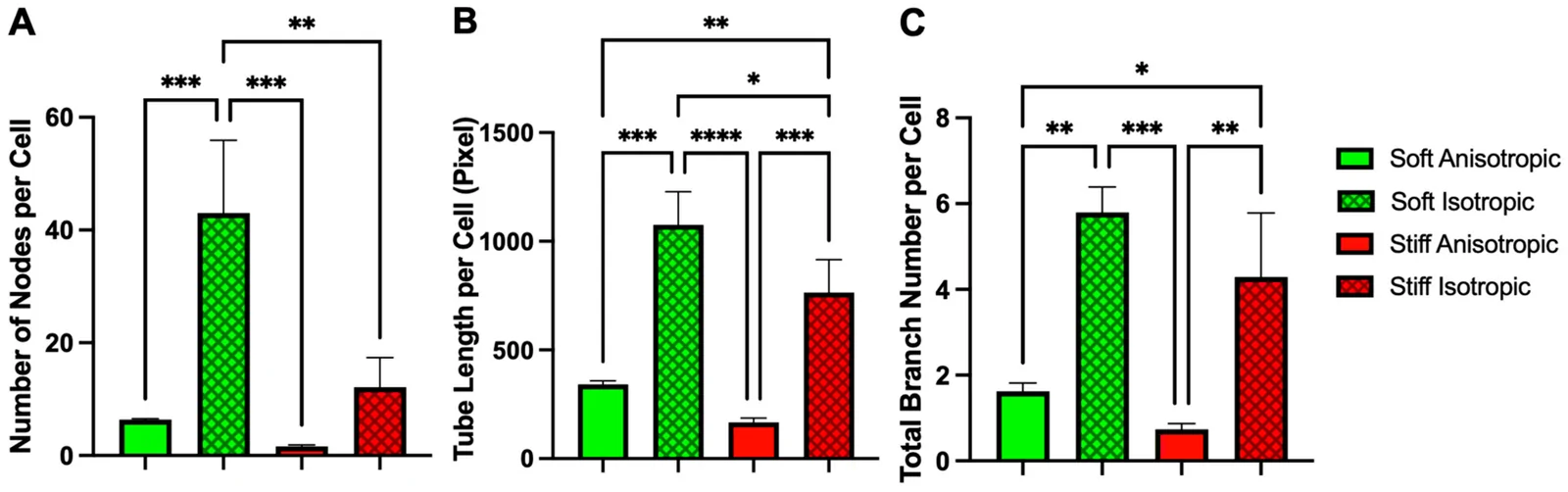
Inhibitory effects of substrate stiffness and anisotropy on the total number of nodes (**A**), total tube length (**B**), and total branch number (**C**) in HUVECs. * *p* < 0.05, ** *p* < 0.01, *** *p* < 0.005, **** *p* < 0.001.

We have observed some similar responses in HUVECs and HCMECs in terms of general cell behavior. Interestingly, when examining the cells’ angiogenic potential by tubulogenesis assay, we observed distinct responses between these cell types. In this section, we first present the results observed in HUVECs. As shown in Figure 4, the angiogenic potential of HUVECs was measured by the number of nodes, total tube length, and the total number of branches. In all these indices, we found similar trends in comparisons. That is, neovessel formation was significantly suppressed by substrate anisotropy when comparing the same stiffness (color) scaffolds. These results show that substrate anisotropy negatively impacts the angiogenic potential of HUVECs.

Furthermore, increased stiffness led to a similar inhibitory effect on the cells. In either fiber alignment condition (anisotropy or isotropy), the stiff scaffold resulted in or tended to result in a reduction in tube formation metrics when compared to the soft scaffold. This effect was stronger in isotropic than anisotropic groups. These findings indicate that substrate stiffening acts as a suppressive factor for HUVEC angiogenesis, limiting their ability to form vascular structures.

### 3.4. Neovessel Formation Is Enhanced by Substrate Stiffening but Suppressed by Anisotropy in HCMEC

**Figure 5:**
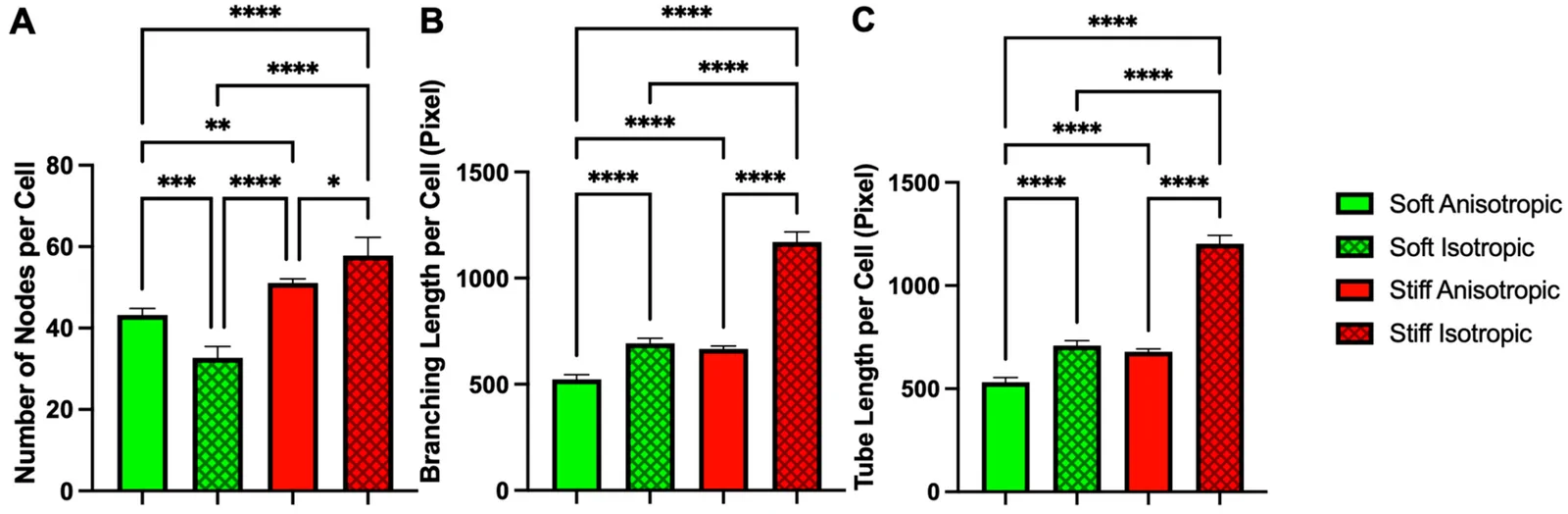
Substrate stiffness enhances the number of nodes (**A**), branching length (**B**), and total tube length (**C**) in HCMECs. Substrate anisotropy inhibits angiogenesis, as measured by these parameters, except for the number of nodes in soft scaffolds (**A**). * *p* < 0.05, ** *p* < 0.01, *** *p* < 0.005, **** *p* < 0.001.

In contrast to HUVECs, HCMECs exhibited a different response to mechanical cues, as there were opposite effects of substrate stiffening and anisotropy on their angiogenic potential. As shown in Figure 5, increased stiffness led to enhanced neovessel formation, which is evident by larger measurements in the number of nodes, total branching length, and total tube length. However, substrate anisotropy had the opposite effect. Comparison of scaffolds with similar stiffness (color) showed a universal reduction in these indices from isotropic (shaded bar) to anisotropic (solid bar) groups. The only exception was the node number in soft scaffolds (Figure 5A), where substrate anisotropy led to an increased number of nodes. These findings suggest a suppressive role of anisotropy in neovessel formation in HCMECs.

### 3.5. Distinct Mechanical Regulations of Protein Expressions Between Two Cell Types

**Figure 6:**
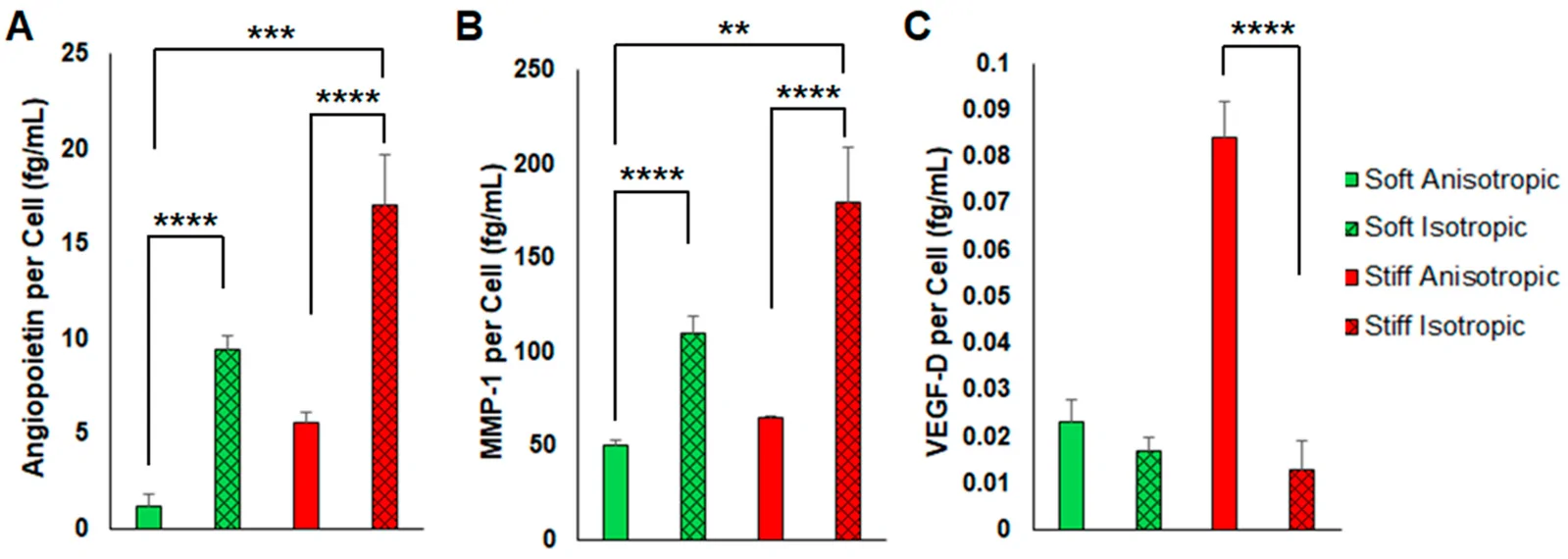
Mechanical regulation of angiopoietin (**A**) and MMP-1 (**B**) expressions in HUVECs and VEGF-D expression (**C**) in HCMECs. ** *p* < 0.01, *** *p* < 0.005, **** *p* < 0.001.

From the multiplex assay, we found different angiogenic protein expression responses to substrate mechanics between HUVECs and HCMECs. In HUVECs (Figure 6A,B), the presence of anisotropy reduced the angiopoietin and MMP-1 expressions in both soft and stiff groups, and stiffening tended to increase the expressions of these proteins, but the changes did not reach statistical significance. The stiff and isotropic group (diseased RV) exhibited higher expressions than the soft and anisotropic (healthy RV) group. These results are consistent with our observation in tubulogenesis results. In HCMECs (Figure 6C), different protein expressions were observed than those from the HUVECs. Substrate anisotropy tended to increase VEGF-D expression in both soft and stiff scaffolds, and the effect was significant in the stiff scaffolds. The substrate stiffness seemed to have different effects between anisotropic and isotropic groups, but the statistical significance was not reached. There was no marked difference in other protein expressions in these cells. The complete panel of angiogenic and endothelial injury protein expressions is reported in the Supplementary Table S1.

## 4. Discussion

In this study, we investigated the impacts of RV-like substrate stiffness and anisotropy on the angiogenic potential of ECs. We found that substrate anisotropy increased cell number and suppressed cell metabolism in both HUVECs and HCMECs, with no notable effect of stiffness on these behaviors. Moreover, we observed cell-type-dependent mechanical regulations on angiogenic potential. HUVECs showed reduced angiogenic potential in response to increased stiffness and anisotropy. In contrast, HCMECs showed enhanced angiogenic potential with substrate stiffening but reduced angiogenic potential with the presence of anisotropy. Therefore, our findings originally demonstrated the role of mechanical cues in EC behavior in the context of RV failure. Our work advances the understanding of mechanobiology in capillary rarefaction and improves the cell-based therapeutic design to restore cardiac performance.

### 4.1. RV-like Mechanics’ Impact on Cell Behavior

Early work on EC response to matrix stiffness is mainly on 2D substrates, since the vascular EC in vivo can be viewed as a 2D structure. In this context, the investigations on EC responses are focused on the morphology and alignment, as well as the function related to inflammation and atherosclerosis formation. Yeh et al. reported that the proliferation of ECs on stiff hydrogels (21.5 kPa) was enhanced compared to soft hydrogels (1.72 kPa) [31]. Andersen Eguiluz et al. found that increased substrate rigidity (40 kPa) enhanced the heterogeneous stress distribution and exacerbated the monolayer integrity when the pulmonary ECs were activated by oscillatory loading [32]. The substrate’s mechanical effect was through the focal adhesion (FA) remodeling. The impairment of EC integrity and increased EC proliferation under a stiff matrix have been observed elsewhere [33,34,35,36,37,38], which are associated with YAP/TAZ activation in EC. A more recent study further identified transcriptome changes in EC modulated by substrate stiffness [39]. The cell responses in soft hydrogel (4 kPa) and stiff tissue culture plate (supraphysiological elasticity) were compared, and cell clusters with endothelial-mesenchymal transition were noted under the stiff substrate condition. A trend of increasing proliferation under a stiff substrate was reported as well. However, to our knowledge, the mechanical control of EC growth is rarely investigated in cardiac-like mechanical environments.

In this study, we adopted the RV-relevant stiffness and anisotropy degree to investigate EC mechanobiology. The measurements of cell counts and metabolic activity showed a minimal impact of substrate stiffness on these cell behaviors, and the observation was consistent between both cell types. In the HUVEC, we even observed an opposite effect in isotropic scaffolds: a stiff substrate led to reduced cell numbers. The substrate elasticity used in our study is much higher than prior work, with the maximal modulus in the range of tens of kPa. Thus, we would first attribute the discrepancy to the distinct mechanical environment used compared to previous studies. Cardiac fibrosis is commonly reported in hypertrophic hearts and occurs in the very early stage of remodeling (days after the onset of pressure overload). Our study suggests that stiffness range should be considered in tissue engineering research as cells’ response can be highly sensitive to matrix elasticity ranges [40].

More importantly, our data demonstrated that substrate anisotropy (rather than stiffness) plays a significant role in modulating these behaviors. Specifically, the presence of anisotropy increased cell number (Figure 2) but simultaneously reduced metabolic activity (Figure 3) in both HUVECs and HCMECs. We speculate that the aligned nanofibers may contribute to the higher cell number through heterogeneous distributions of intracellular stress and cell–matrix interactions along the preferred fiber alignment. Since the in vivo EC morphology is typically in an elongated shape, the cells may adapt better in the heterogeneous rather than homogeneous stress environment. The high cell number, if resulting from increased cell proliferation, may explain the opposite trend found in cells’ metabolic activity. During high proliferation, cells tend to downregulate oxidative phosphorylation, which is a more efficient but slower process for generating ATP [41,42]. Thus, our hypothesis is that the metabolic switch allows cells to quickly produce the necessary components for cell division, but it results in lower overall metabolic activity. While these are possible explanations of our results, one cannot rule out other causes such as cytoskeletal organization, mechanotransduction pathways, or YAP/TAZ signaling. Future work may further investigate the underlying mechanobiological mechanisms.

### 4.2. RV-like Mechanics’ Impact on EC Angiogenic Potential

Prior 2D studies have reported that increased matrix stiffness led to enhanced neovessel branching [36,38]. Particularly, Bordeleau et al. demonstrated that angiogenesis was enhanced by the mechanical stiffness of the collagen gels, not the matrix density [36]. Moreover, the angiogenic outgrowth depends on MMP activity. Interestingly, using gelatin-based 3D colloidal gels, Nair et al. reported the opposite observation that matrix stiffening reduced the extent of EC capillary network [43], which is consistent with other 3D hydrogel studies [44,45]. The first explanation for the discrepant observations may be attributed to the 3D vs. 2D microstructural environment. But all these studies have acknowledged that the increase in stiffness in these 3D substrates is likely due to increased matrix density. This factor outweighs the mechanical stiffness’s influence and prevents solute diffusion and proteolytic activities, which are essential for angiogenesis. In our study, cell filtration from the top surface into the PEUU scaffolds was observed as the thickness of the scaffolds varied from 0.15 mm to 0.22 mm. We have also examined the porosity of the PEUU scaffolds, and they are identical across the groups [27]. Thus, the mechanical response should be restricted to the matrix stiffness and anisotropy degree.

Using the RV-like substrate stiffness and anisotropy, we found that HUVECs and HCMECs exhibited distinct angiogenic responses when exposed to varying mechanical properties. In HUVECs, both increased stiffness and anisotropy were associated with reduced neovessel formation. In contrast, in HCMECs, increased stiffness enhanced angiogenesis, whereas anisotropy suppressed it. The suppressive role of both mechanical factors for HUVECs resulted in the lowest angiogenic capacity in the stiff and anisotropic group (Figure 4), which represents the pressure-overloaded RV mechanical properties. This indicates that pathological remodeling in vascular tissues may negatively affect EC homeostasis. However, the trend in the HCMECs was the opposite: the cells from the stiff and anisotropic group exhibited higher angiogenic capacity than those from the soft and anisotropic group (Figure 5). Thus, it implies that a pathological mechanical environment can potentially favor angiogenesis in this cell type. A further examination of the protein expressions in these two cell types (Figure 6) confirmed distinct responses of HUVECs and HCMECs to the mechanical cues.

The differential regulations by mechanical cues underscore the importance of using anatomically relevant cell lines in mechanobiological studies of the cardiovascular system. While HUVECs are the most used type of ECs in the field, our results suggest that one should be cautious in translating findings from HUVECs for cardiac research. On the other hand, HCMECs are microvascular—not arterial—endothelial cells, and future studies may confirm our findings with ECs collected from arteries. The use of substrates with varying stiffnesses of healthy and pathological RVs will be necessary in identifying specific biomolecular targets in ECs to reverse or halt maladaptive responses in RV failure progression.

### 4.3. Implications in Capillary Rarefaction in RV

As HCMECs originate from the coronary circulation, including both macro- and micro-circulation vascular ECs, their responses may be more relevant to RV failure, and we will mainly discuss the implications of findings from this cell type. It is known that pressure-overload-induced remodeling results in both tissue stiffening and enhanced anisotropy. Our data suggest that the combined effect of hypertensive remodeling (stiff and anisotropic) led to elevated angiogenic capacity of EC compared to the healthy RV’s mechanical environment (soft and anisotropic) (Figure 5). The pro-angiogenic impact of matrix stiffness overcomes the anti-angiogenic effect of anisotropy, and the overall EC response might favor capillary angiogenesis. Moreover, the EC’s angiogenic potential was the largest in the stiff and isotropic group (Figure 5). While there is a lack of mechanical data for ischemic RVs, it is reasonable to assume that, like the left ventricle, the loss of cardiomyocytes and formation of scarring tissue in this region would result in a stiffer and isotropic matrix environment. Our result thus indicates that the EC may respond to such adverse remodeling by releasing more pro-angiogenic factors, which is a beneficial effect. This is similar to the observation in tumor progression that larger stiffness in tumor tissue often promotes angiogenesis, although the outcome is detrimental in the scenario of tumor tissue. Finally, our data do not fully reveal insights into the development of capillary rarefaction, and a marked distinction exists between observations from simplified in vitro scaffold models and those from complex in vivo pathological remodeling. Microvascular dysfunction in hypertensive hearts is one of the leading causes of angina in heart diseases, and the pathogenetic mechanisms are still largely unknown [46]. Additionally, the improvement of capillary rarefaction has been reported with some commonly used therapeutics in the management of heart failure, such as angiotensin-converting enzyme inhibitors (ACEi) and sodium glucose transport inhibitors (SGLT-2) [47,48]. Other strategies under development include the use of growth factors, cells, extracellular vesicles, and non-coding RNAs to target the endothelium and enhance neovessel growth [49]. However, the degree of enhanced capillarization with these therapeutics at varying wall stiffnesses is unknown and should be examined to optimize dosing regimens. Further study can investigate the impact of RV-specific mechanisms on other EC functions (e.g., pro-atherosclerotic, pro-inflammatory).

### 4.4. Limitations

There are a few limitations in the present work. First, we measured the total number of cells as an indirect measure of cells’ pro-angiogenic function (EC growth). But these data could result from multiple factors such as enhanced proliferation, altered attachment efficiency, or reduced apoptosis. The exact mechanism associated with the changes in cell number was out of the scope of this study and will be investigated in future work. Moreover, the use of HCMECs includes ECs from both proximal arterial and distal capillary sites, which may exhibit different phenotypes but are ignored in the present work. As micro-circulation ECs are more relevant to RV capillary rarefaction and no commercially available coronary capillary ECs, we chose this cell source in the present work. The heterogeneity of ECs in the coronary circulation can be elucidated next. Third, we used one-way ANOVA as the statistical method because the primary objective was to compare the four pre-defined scaffold conditions (associated with physiologically relevant states) as integrated mechanical environments. We acknowledge that a more comprehensive mechanobiology study could incorporate more stiffness or anisotropy ranges in the scaffold groups and adopt two-way ANOVA methodology. Similarly, because HUVECs and HCMECs have different biological baselines, the two cell types were analyzed separately, and no statistical analyses were performed to compare between cell lines. Fourth, the present work only investigated a single cell type (EC) in the RV-like mechanical environment. A more comprehensive assessment of the in vitro scaffold model can be obtained with the presence of cardiomyocytes, fibroblasts, smooth muscle cells, immune cells, or extracellular matrix remodeling.

## 5. Conclusions

In the present work, we demonstrated the individual effects of RV-like substrate stiffness and anisotropy on the cell behavior of HUVECs and HCMECs, with a focus on the angiogenic potential. Our findings highlight the importance of incorporating both matrix mechanical properties and the origin of the EC line in cardiovascular research. For HUVECs, neovessel formation is suppressed by substrate stiffening and anisotropy; whereas for HCMECs, substrate stiffening enhances endothelial cell angiogenic potential and anisotropy has the opposite effect. These results have potential implications for developing new biomaterials or in vitro models to promote angiogenesis and improve cardiac function in patients with RV failure.

## Data Availability

Research data are available for sharing upon requests to the corresponding author.

## Supplementary Materials

The following supporting information can be downloaded at: https://www.mdpi.com/article/10.3390/bioengineering13080849/s1. Supplementary Table S1. Endothelial secretome profiling quantified from the multiplex assay on the conditioned media collected in ECs at 72 hr of culture on scaffold groups or blank wells (medial control). The protein expression (per cell) results (in femtogram/mL) are shown as Mean ± SD. n = 3 per group. N/A indicates non-detectable proteins. VEGF-R2: vascular endothelial growth factor—receptor 2, also known as KDR or Flk-1; VEGF-R3: vascular endothelial growth factor—receptor 3, also known as FLT-4; VLA-4: very late activation antigen-4, also known as α4β1 integrin; CD62P: P-Selectin; CD62E: E-Selectin; MMP-1: matrix metalloproteinase-1; TM: Thrombomodulin; VEGF-A: vascular endothelial growth factor-A; VEGF-D: vascular endothelial growth factor-D.
