# Supplementary material for: Angiogenic Potential of Endothelial Cells in Response to the Stiffness and Anisotropy of Right Ventricle Mimetic Scaffolds
Source: Bioengineering (Basel). 2026 Jul 23;13(8):849. doi: 10.3390/bioengineering13080849 (PMC13510070; doi:10.3390/bioengineering13080849)
Supplement: Supplementary file 1 [file bioengineering-13-00849-s001.zip › bioengineering-4409696-supplementary.pdf]

Supplementary Table

Table S1. Endothelial secretome profiling quantified from the multiplex assay on the conditioned media collected in ECs at 72 hr of culture on scaffold groups or blank wells (medial control). The protein expression (per cell) results (in femtogram/mL) are shown as Mean ± SD. n = 3 per group. N/A indicates non-detectable proteins. VEGF-R2: vascular endothelial growth factor – receptor 2, also known as KDR or Flk-1; VEGF-R3: vascular endothelial growth factor – receptor 3, also known as FLT-4; VLA-4: very late activation antigen-4, also known as α4β1 integrin; CD62P: P-Selectin; CD62E: E-Selectin; MMP-1: matrix metalloproteinase-1; TM: Thrombomodulin; VEGF-A: vascular endothelial growth factor-A; VEGF-D: vascular endothelial growth factor-D.

	**HUVEC**				
		**A**	**B**	**C**	**D**
	**Media Control (Background)**	**Soft & Anisotropic**	**Soft & Isotropic**	**Stiff & Anisotropic**	**Stiff & Isotropic**
**Angiopoietin**	N/A	1.2±0.06	9.4±0.8†	5.6±0.1*	17±2.7$^{\dagger,\#}$
**CD62P**	2.0 ± 0.5 (x10^{6})	13±0.64	8.8±0.8†	45±0.4	42±6.8
**VEGF-R2**	84 ± 25 (x10^{3})	1.0±0.05	1.2±0.1†	1.8±0.02	11±1.8†
**VEGF-D**	3.2 ± 2.5 (x10^{3})	0.04±0.002	0.01±0.001†	0.07±0.001	0.5±0.09†
**MMP-1**	102 ± 42 (x10^{3})	50±3	110±9†	65±1*	180±29$^{\dagger,\#}$
**CD31**	N/A	78±3.9	120±10†	64±0.60	210±33†
**TM**	61 ± 13 (x10^{3})	1.1±0.1	2.4±0.2†	1.7±0.02	11±1.8 †
**VEGF-R3**	N/A	N/A	N/A	N/A	N/A
**VLA-4**	138 ± 66 (x10^{3})	N/A	N/A	N/A	N/A
**VEGF-A**	18±1 (x10^{6})	N/A	N/A	N/A	N/A
**CD62E**	NA	N/A	N/A	N/A	N/A
**Syndecan**	4.6 ± 1.0 (x10^{3})	N/A	N/A	N/A	N/A
	**HCMEC**				
	**Media Control (Background)**	**Soft & Anisotropic**	**Soft & Isotropic**	**Stiff & Anisotropic**	**Stiff & Isotropic**
**VEGF-D**	3.2 ± 2.5 (x10^{3})	0.023±0.004	0.017±0.003	0.084±0.008*	0.013±0.006†
**MMP-1**	102 ± 42 (x10^{3})	14±3	14±3	16±1	38±16
**VEGF-R2**	84 ± 25 (x10^{3})	N/A	N/A	N/A	N/A
**VLA-4**	137 ± 66 (x10^{3})	N/A	N/A	N/A	N/A
**Angiopoietin**	N/A	N/A	N/A	N/A	N/A
**VEGF-R3**	N/A	N/A	N/A	N/A	N/A
**VEGF-A**	18±1 (x10^{6})	N/A	N/A	N/A	N/A
**Syndecan**	4.6 ± 1.0 (x10^{3})	N/A	N/A	N/A	N/A
**CD31**	N/A	N/A	N/A	N/A	N/A

**TM**	60 ± 12 (x10^3)	N/A	N/A	N/A	N/A
**CD62E**	N/A	N/A	N/A	N/A	N/A
**CD62P**	2.0 ± 0.5 (x10^6)	N/A	N/A	N/A	N/A

*$p<0.05$ soft vs. stiff scaffolds (A vs C or B vs D); †$p<0.05$ anisotropic vs. isotropic scaffolds (A vs B or C vs D); #$p<0.05$ vs. soft & anisotropic scaffold (vs. A).
